# Fear, Fanaticism, and Fragile Identities

**DOI:** 10.1007/s10892-023-09418-9

**Published:** 2023-01-26

**Authors:** Ruth Rebecca Tietjen

**Affiliations:** Department of Philosophy, Tilburg University, Warandelaan 2, 5037 AB Tilburg, The Netherlands

**Keywords:** Fanaticism, Sacred values, Fear, Anxiety, Uncertainty, Fraternity-terror, Social identity

## Abstract

In this article, I provide a philosophical analysis of the nature and role of perceived identity threats in the genesis and maintenance of fanaticism. First, I offer a preliminary definition of fanaticism as the social identity-defining devotion to a sacred value that demands universal recognition and is complemented by a hostile antagonism toward people who dissent from one’s group’s values. The fanatic’s hostility toward dissent thereby takes the threefold form of outgroup hostility, ingroup hostility, and self-hostility. Second, I provide a detailed analysis of the fears of fanaticism, arguing that each of the three aforementioned forms of hostile antagonism corresponds to one form of fear or anxiety: the fanatic’s fear of the outgroup, renegade members of the ingroup, and problematic aspects of themselves. In each of these three forms of fear, the fanatic experiences both their sacred values and their individual and social identity as being threatened. Finally, I turn to a fourth form of fear or anxiety connected to fanaticism, namely the fanatic’s anxiety of and flight from the existential condition of uncertainty itself, which, at least in some cases, ground the fanatic’s fearfulness.

## Introduction

Fanatical intolerance, hostility, and violence are pressing problems in the contemporary world. Curtailing and confronting them requires us to deepen our understanding of what fanaticism is. In this paper, I aim to contribute to this endeavor by exploring the role of fear, anxiety, and uncertainty in the genesis and maintenance of fanaticism.

Intuitively, we can distinguish between a broad and a narrow notion of fanaticism. At the heart of any form of fanaticism is the identity-defining devotion to an object or idea that is pursued with extraordinary fervor. According to the broad understanding of the term “fanaticism,” these two features—devotion and fervor—are sufficient to make someone a fanatic. Fanaticism, broadly construed, accordingly encompasses both what we can call “coercive” and “non-coercive” forms of fanaticism (for a similar distinction, see Passmore 1990: 5–6). Non-coercive fanaticism includes phenomena such as sports fandom (see, e.g., Tarver 2017), consumer fanaticism (see, e.g., Chung et al. 2018; Fuschillo 2020), and vocation. They require devotion, may demand sacrifices, and harm others or oneself, but they do not come with a demand for the universal recognition of one’s values, let alone with the attempt to impose one’s values and way of life on other people as is characteristic of coercive forms of fanaticism. In fandom, one’s own and one’s group’s identity is defined against that of outsiders, observers, consumers, and fans with other loyalties. If everybody were a fan of the same team, artist, or brand, fandom could not fulfill its individualizing function, and the phenomenon of vocation would disappear if everyone had the same vocation.

When I am talking about fanaticism in this article, I am talking about fanaticism, narrowly construed—i.e., coercive forms of fanaticism in which (as I will elaborate in Sect. 2) the identity-defining devotion to an object or idea comes with the demand for the universal recognition of one’s values and is complemented by a hostile antagonism toward people who dissent from one’s values (Katsafanas 2019; Passmore 1990). Fanatics of this kind not only fervently pursue their goals; they also fervently fight those who do not share their values. As an example, one might think of a Christian anti-abortionist who is passionately committed to the sacred value of human life, requires the universal recognition of their value, and, in the most extreme case, murders doctors who carry out abortions in order to protect what they take to be sacred (Olson 2011). Fanatics of this kind aim to eliminate dissent (by eliminating dissenters) and yet they also need these “others” whom they aim to eliminate to uphold their sense of identity; their identity is dialectically interwoven with that of their enemies (Katsafanas 2022; Szanto 2020). When I am talking about fanaticism in this paper, I am talking about fanaticism in this narrow, political sense.

Fanaticism, narrowly construed, accordingly involves both devotion and hostile antagonism. But what is it that binds these two complementary components together? One important element is fear and anxiety. As noted in the contemporary philosophical discourse, fanatics fear dissent; they are hostile toward people who threaten their values and, in threatening their values, threaten their individual and social identity. For example, Katsafanas (2019: 15) argues that fanatics “see the status of their own values as threatened by the absence of widespread acceptance” and, with their values, they experience their group’s identity and existence as threatened. Thomas Szanto (2022: 194, 202) claims that “[t]he formal object of fanaticism [i.e., that which distinguishes fanaticism from other affective phenomena] is the *threat* to the sacred value issuing from harm, questioning or dissent”; moreover, fanaticism also “essentially *reinforces* the perception of threat” by making the fanatic over-concerned with their threatened self-respect.

Similar claims about the role of fear, anxiety, and uncertainty can be found in the literature on other hostile political movements and sentiments including those of extremism, fundamentalism, populism, and group-centered hatred. For example, Berger (2018: 150, 112, my emphasis) describes how “feelings of uncertainty” contribute to the emergence of extremism and identifies the construction of an “out-group as an intrinsic *threat* to in-group legitimacy” as a defining feature of extremism. In a similar spirit, fundamentalism has been characterized as “a strategy by which *beleaguered believers* attempt to preserve their distinctive identities as individuals or groups in the face of modernity and secularization” (Ruthven 2007: 5–6, my emphasis). Mikko Salmela and Christian von Scheve (2017: 567, my emphasis; see also Salmela and von Scheve 2018) describe how in right-wing populism, through the affective mechanism of *ressentiment*, “insecurities [that] manifest as *fears* of not being able to live up to salient social identities and their constitutive values” are “transformed through repressed shame into anger, resentment, and hatred towards perceived ‘enemies’ of the self and associated social groups.” Vendrell Ferran (2021, Sect. 3, my emphasis), finally, argues that “a *threat* to one’s self-worth” is a crucial element of the developmental history of both individual and group-centered hatred.

In short, all these authors refer to phenomena of fear, anxiety, and uncertainty that in one way or another reflect perceived threats to one’s values and identity. However, they do not offer us a fine-grained account of the exact nature of fear and anxiety in this context. Especially, although the experiences in question are sometimes identified as affective, they are usually not explored from the perspective, and with the tools, of philosophy of emotions (for an important exception, see Nussbaum 2018). This is true even of the aforementioned literature within philosophy of emotions that explores the phenomena of fear and anxiety and yet focuses on other affective phenomena such as hatred (Vendrell Ferran 2021), *ressentiment* (Salmela and von Scheve 2017; Salmela and Capelos 2021), or the affective mechanism of fanaticism itself (Szanto 2022). This leaves our understanding of fanaticism incomplete because we lack a comprehensive account of

1. exactly what kinds of fear-like phenomena we are dealing with in this context (e.g., fear, anxiety, or uncertainty);
2. what kinds of objects these feelings specifically target (e.g., an abstract process such as modernization, a person or group of persons such as an outgroup, renegade members of the ingroup, or problematic aspects of oneself such as one’s own inner ambiguity);
3. what exactly it is that is taken to be under threat (e.g., one’s descriptive or evaluative self-conception, one’s individual or social identity, one’s self-worth or existence); and
4. how the different dimensions and phenomena of fear, anxiety, and uncertainty interact.

This article seeks to fill this lacuna by providing an analysis of the nature and role of perceived identity threats in the genesis and maintenance of fanaticism and uncovering the self-relationship underlying the fears of fanaticism. In my analysis, I draw on philosophical literature as well as findings from neighboring disciplines, especially social psychology, sociology, and political theory.

The aim and perspective of my article are limited in a threefold way. First, I focus on the phenomenon of fanaticism rather than on other hostile political movements and sentiments. I hypothesize that due to structural similarities, my analysis is also relevant to our understanding of (some of) the other abovementioned phenomena. The investigation of this claim, however, would require a comprehensive analysis of the similarities and differences between different antagonistic political movements and sentiments, which exceeds the scope of the present article (see, e.g., Cassam 2021 for an attempt to determine the relationship between extremism, fanaticism, and fundamentalism).

Second, I bracket the question of whether besides fear, anxiety, and uncertainty, other affective phenomena, such as envy, shame, humiliation, and *ressentiment*, are relevant to understanding the role of perceived identity threats in the present context (see, for example, Katsafanas 2022; Salmela and Capelos 2021; Scheler 2017). My claim is that in order to understand fanaticism, we need to understand its relation to fear and anxiety, and in order to understand the role of fear and anxiety in fanaticism, we need to appreciate how fear and anxiety engage the fanatics’ identity. Contrary to Nussbaum (2018), however, I claim neither the primacy of fear over other emotions in understanding and explaining fanaticism nor that fear is necessarily anti-social or anti-democratic (for other, more nuanced views on the political role of fear and anxiety, see, for example, Kurth 2018; Robin 2006; Shklar 1989).

Third, the article does not involve any reflections on the history and socio-political embeddedness of the concept and phenomenon of fanaticism (see, e.g., Cavanaugh 2012; Colas 1997; Goldsmith 2022; Spaemann 1971; Toscano 2017). This is a pragmatic decision due to the limited space and systematic interest of this article. That said, I fully acknowledge the importance of such considerations, especially in light of the normative dimension and often discrediting function of the concept of fanaticism in public discourse. Applied to the present context, a more comprehensive analysis of “the fears of fanaticism” would need to complement the analysis of *the fears that fanatics have* with an analysis of *the fearful attitudes toward fanaticism* that might underlie and ground the pejorative usage of the term “fanaticism.” While the expression “the fears of fanaticism” allows for both readings (as *genitivus subjectivus* and *genitivus objectivus*), here I foreground the first reading, while only hinting at the second one at the very end of my analysis.

The structure of the article is as follows. In Sect. 2, I offer a preliminary definition of fanaticism as a compound of devotion and hostile antagonism. In Sect. 3, I provide a detailed analysis of the role of fear and anxiety in the genesis and maintenance of fanaticism, thereby distinguishing fear of the outgroup, fear of renegade members of the ingroup, and self-reflexive anxiety as three dimensions of the fanatic’s fearfulness. In Sect. 4, I introduce a fourth form of fear or anxiety that is connected to fanaticism and that, at least in some cases, grounds the fanatic’s fearfulness, namely the fanatic’s anxiety of and flight from the existential condition of uncertainty itself. Section 5 offers an outlook that takes up the issue of fearful attitudes toward fanaticism and highlights the importance of foregrounding the liberatory potential of uncertainty and ambiguity for preventing fanaticism.

## What is Fanaticism?

As outlined in the introduction, at the core of fanaticism is the social identity-defining devotion to a sacred value that is defended and realized with extraordinary pursuit (Katsafanas 2019, 2023; see also Hoffer 2019). A “sacred value” here is meant to be an infinite value that cannot be traded off against other values (see, e.g., Baron and Spranca 1997; Ginges and Atran 2014; Tetlock 2003). The above-mentioned anti-abortionist, for example, treats human life as sacred. While even the mere consideration of trading it off against profane (finite) values causes affective reactions such as indignation and disgust, the necessity of “trade-off” against other sacred values is experienced as tragic (Katsafanas 2023). Despite its religious connotation, in social psychology the concept of sacred values is used to denote both religious and non-religious values. The term “fanaticism” accordingly is not restricted to religious phenomena.

The devotion to the sacred value is identity-defining—i.e., it is part of the fanatic’s practical self-conception (on the concept of practical self-conception, see Frankfurt 1988; Korsgaard 1996; Velleman 2002). More precisely, the value attachment in question is part of both the fanatic’s descriptive and evaluative self-conception. As a part of the fanatic’s descriptive self-conception, the value attachment is part of who the fanatic takes themselves to be (and who others take them to be). As a part of the fanatic’s evaluative self-conception, it is a description under which the fanatic values themselves. Losing or abandoning the affective attachment in question therefore would both impair the fanatic’s (valuable and/or valued) capacity to understand themselves and diminish their self-esteem. This explains why the fanatic—like many of us—tends to experience challenges to their self-conception as aversive even though changing or fragmenting one’s identity in itself is neither good nor bad and might be seen as a precondition of personal growth (Tietjen 2020). To summarize, in the terms of contemporary philosophy of emotion, we can say that the fanatic’s attachment to their sacred value is a passion or, as it is sometimes called, “sentiment” (see Roberts 2007: 17–22 on the concept of passions, and Ben-Ze’ev 2000: 82–86 on the concept of sentiment)—i.e., an affective attachment to an object that is constitutive of the person’s practical identity and bestows their life with continuity, coherence, and/or meaning (Tietjen 2021a).

Moreover, fanaticism essentially is a group phenomenon (Crosson 2003; Hoffer 2019: 113–157; Marcel 2008). It is both the fanatic’s individual identity and the identity of the fanatical group that is defined through the shared passionate commitment to a sacred value (Katsafanas 2019). The fanatic’s passionate commitment accordingly binds them to both their object of passionate devotion and a community of fellow fanatics. As their manifestos reveal, even lone-actor terrorists, such as Anders Breivik and Brenton Tarrant, claim to act in the name of such a (real or imaginary) group (see Katsafanas 2022; Szanto 2022).

The fanatical group’s joint passionate commitment to a sacred value is complemented by their hostile antagonism toward people who do not share their values and, therefore, threaten their group’s values, identity, and existence. As I explain in more detail below, this antagonism toward dissent takes a threefold form:

1. First, there is a hostile antagonism toward the outgroup—those who are *not* committed to the fanatical group’s sacred values (outgroup hostility).
2. Second, there is a hostile antagonism toward renegade members of the ingroup—i.e., those fellow fanatics who fail to properly follow the fanatical group’s code of conduct and do not feel, think, behave, and act how they ought to feel, think, behave, and act as members of the fanatical group (ingroup hostility).
3. Third and finally, there is a hostile antagonism toward problematic aspects of oneself—i.e., toward any of one’s own features and states (feelings, desires, cognitions, behaviors, actions) that indicate that oneself might not be fully committed to one’s group’s sacred values (self-hostility).

Fanatics pursue their goals fervently. In their struggle for what they take to be good, against what they take to be evil, the fanatical group is willing to make use of unconventional means—i.e., means that violate the existing social, moral, political, or legal order (Olson 2007). Most importantly, they are willing to sacrifice their own or others’ well-being or lives. Here, I am consciously talking about the fanatical group rather than the individual fanatic who may or may not (be willing to) actively engage in violence. (Self-)Sacrifice thereby, on the one hand, answers to the call of the sacred; the sacred demands us to sacrifice all our other interests, including our self-interest, for the sake of the infinitely more valuable good (Tietjen 2021b). On the other, sacrifice has a performative dimension. As underlined by the etymology of the term, sacrifice sets apart the sacred from the profane and, in doing so, “makes” one’s object of devotion sacred. At the same time, the fanatical individual and group are purified through sacrifice; even if, on the psychological level, they still “have” other, profane values and interests, they act as if the latter do not matter and, in doing so, silence not only outer dissent but also inner ambiguity (on both the individual and group level).

## The Fears of Fanaticism

In the previous section, I introduced the phenomenon of fanaticism and argued that it involves, first, a joint passionate commitment to a sacred value that is pursued fervently and, second, a hostile antagonism toward those people who do not share the fanatical group’s values and, therefore, threaten their group’s values, identity, and existence. In this section, I provide a detailed analysis of the fears of fanaticism. In a nutshell, my claim is that each of the three aforementioned forms of hostile antagonism—outgroup hostility, ingroup hostility, and self-hostility—corresponds to one form of fear or anxiety: fear of the outgroup, fear of renegade members of the ingroup, and fear of problematic aspects of oneself. In each of these three forms of fear directed toward the possibility or reality of dissent, the fanatic experiences both their sacred values and their group’s identity and existence as threatened.

When talking about “the fears of fanaticism”—i.e., in this context, the fears that fanatics have—I use the concept of fear as an umbrella term denoting all kinds of affective phenomena belonging to the fear family (e.g., fear, anxiety, uncertainty). What they all have in common is that they are affective reactions to aversive possibilities motivating avoidance (see, e.g., Roberts 2003: 193–195). The affective evaluation thereby is dependent on the person’s concerns, that is, on what they care about and attribute worth and value to (see Roberts 2003: 141–151). If, for example, I feel threatened by the possibility of violating my fanatical group’s code of conduct, this might be because I care about my group’s sacred values, identity, and existence that I experience as threatened by my renegade behavior; or it might be because I care for my well-being and life which I experience as threatened through my fellow fanatics who might punish me for my norm violations; or both (which is most likely, as I argue below). The threats in question can be more or less (un)certain and (un)specific. Moreover, they can arise from within or without oneself or one’s group. Accordingly, when I talk about the “fears of fanaticism,” this includes both non-self-reflexive and self-reflexive forms of fear and anxiety. I can be afraid of “the other” who seemingly threatens my own or my group’s values, identity, or very existence but also of my or my group’s misbehaviors and failures.

In the course of my analysis, I turn from more paradigmatic (non-self-reflexive, specific, certain) fears and threats such as the fanatics’ fear of the outgroup to less paradigmatic (self-reflexive, unspecific, uncertain) fears and threats, such as the fanatic’s fear of their fellow fanatics, themselves, and uncertainty in general. In other words, I turn from what (in ordinary language and the jargon of existential philosophy) is sometimes called “fear” (in a narrow sense) to “anxiety.” I follow the conceptual convention of calling the latter phenomena anxiety. However, it is important to keep in mind that the distinction between fear (in a narrow sense) and anxiety is controversial and ambiguous (Tietjen 2019). For this reason, in the present context, I prefer to use more precise concepts to name and differentiate different phenomena of fear and anxiety, such as “self-reflexive anxiety” or “aversive uncertainty.”

### Fear

It is a key feature of fanaticism as well as of other antagonistic political movements and sentiments, such as extremism, populism, and group-centered hatred, that they involve a form of group antagonism. This group antagonism involves the construction of a morally laden frontier between a relatively homogenous ingroup and an outgroup (see Katsafanas 2019: 15–17 on fanaticism; Berger 2018: 51–74 on extremism; Mudde and Rovira Kaltwasser 2017: 1–20 on populism; and Szanto 2020 on group-centered hatred).

Ingroup–outgroup demarcations in the context of fanaticism display two characteristic features. First, whereas the question of who does and who does not belong to an in- or outgroup is usually subjective, contestable, context-dependent, and may change over time, fanatics are particularly concerned with *clear* ingroup–outgroup demarcations (see, e.g., Berger 2018: 52–54 for a similar claim on extremism). Fanatical groups have what social psychologists call a high “entitativity” (Campbell 1958)—i.e., they are “clearly structured … with sharp boundaries, unambiguous membership criteria, highly shared goals, and consensus on group attributes” (Hogg 2007: 88). At least the ingroup, and in some cases also the outgroup, is construed as relatively homogenous. Since homogeneity is a matter of perspective—it is dependent on which aspects one takes into consideration—clear definitions of group affiliations usually come with a focus on specific aspects of identity rather than a comprehensive view of who oneself and the other is. Although in principle, all aspects of identity can serve as criteria to define group membership, some are more suitable than others to establish boundaries that are both clear and unalterable. This explains the propensity of fanatical and extremist movements to rely on essentialist interpretations of identity (see Hogg 2007: 88; see also Salmela and von Scheve 2017: 585 for a similar claim about right-wing populism).

Second, ingroup–outgroup demarcations need not involve any explicit normative or political dimension. For example, being or not being a member of a swimming club is not a political question (although there may be a political dimension to it, given that swimming clubs are social institutions). In the case of fanatical and other antagonistic political movements, both the normative and political dimensions are crucial. First, the outgroup is held morally responsible for the grievances of the ingroup. This explains why fanaticism and other antagonistic political movements typically involve moral emotions, such as anger, resentment, and indignation. Moreover, in political terms, the ingroup is claimed to be more legitimate than the outgroup. For example, populists blame “those in power” for their failure to fulfill what they take to be legitimate popular demands; they construe “the elite” or “establishment” that they oppose as corrupt and defend the principle of popular sovereignty (Laclau 2007: 76–128). When I am talking about the political dimension of fanatical groups here, I am using the term “political” in a broad sense, referring to the aspiration to determine, dictate, and enforce the norms and rules governing a specific set of social practices. In this regard, political aspirations can be ascribed even to fanatical groups that do not pursue political power in a narrower sense of, e.g., governance.

As the example of populism illustrates, a morally laden frontier between a relatively homogenous ingroup and an outgroup is not enough to make a group fanatical; not every populist party or movement is fanatical. An additional requirement is outgroup hostility. This hostility, in turn, is frequently explained by fear and more specifically the perception of the outgroup as an intrinsic threat to the ingroup (Berger 2018: 112). We are here pointed to the first, most visible form of fear connected to fanaticism, namely the fear of the outgroup that doubts, dissents, or deviates from the fanatical group’s sacred values (Katsafanas 2019). Most poignantly, Thomas Szanto (2022: 194) claims that “[t]he formal object of fanaticism is the *threat* to the sacred value issuing from harm, questioning or dissent” from the outgroup; not only that, fanaticism also “essentially *reinforces* the perception of threat” by generating an over-concern with one’s threatened self-respect.

The fanatic is afraid of dissent—in this case, dissent embodied by members of the outgroup who question, violate, or do not share the fanatical group’s sacred values—because they perceive dissent as a threat to their sacred values. More precisely, they do not simply perceive dissent as a threat to the *realization* of their values; they also perceive the *status of their values as sacred* as being endangered by the lack of widespread acceptance (Katsafanas 2019). Since for the fanatic, their value attachment is not contingent but constitutive of their identity, threats to what they take to be sacred are *simultaneously* experienced as threats to themselves. Since the value attachment in question is constitutive not only of the fanatic’s individual identity but also of their social identity and the identity of the fanatical group with which they identify, threats to the fanatic’s individual identity are *simultaneously* experienced as threats to their social identity and the identity and existence of their group. On the epistemic level, this means that when the fanatic’s values are put into question, their (understanding of their) individual and social identity is put into question. On the practical (ethical) level, this means that the fanatic experiences disrespect for what they take to be sacred simultaneously as disrespect for themselves. This can be illustrated by the phenomenon of so-called “hurt religious sentiments.” What is hurt in this case are not feelings, literally speaking; it is the believer themselves who feels misrecognized through the misrecognition of what they take to be sacred. Since their attachment to the sacred value in question is affective in nature, this violation can be described as a violation of religious sentiments. Since the attachment is constitutive of the believer’s identity, it is a violation of religious sentiments (or passions) rather than, say, emotions. To summarize, it is both the values and identity of the fanatical individual and group that are experienced as put into question through dissent; it is both the sacred object and the fanatical individual and group that are perceived as misrecognized and violated when the fanatical group’s sacred values are questioned or harmed.

To elucidate what is special about the fanatic’s form of passionate devotion and their sense of identity, it is helpful to contrast it with other forms of devotion. First, the fanatic conceives of their value as universal or at least demands that it is recognized by everyone (Katsafanas 2019; Passmore 1990; Tietjen 2021a). This can be contrasted with other forms of passionate devotion such as romantic love, in which our value commitment is highly personal and particular. We do not demand that everyone loves the person we are romantically engaged with in the same way we do. On the contrary, for people who claim that love comes with a demand for exclusivity, the possibility that everyone—or just *anyone*—might love their beloved in the same way they do is highly threatening. However, even if, in the case of romantic love, we do not demand that others adopt the same affective attitude toward the object of our passionate devotion, at least in some cases we may care about other people’s judgment about the *lovability* of our beloved. If someone we trust puts into question our beloved’s lovability, this may seriously disturb our love and shake our sense of identity that is partly defined by our loving relationship. Moreover, we may demand that others treat the person we love with respect.

Second, the fanatic’s emotions reveal a particular form of self-centeredness. At least some forms of love and passionate devotion are “selfless” in the sense that when someone harms the person or object we love or fails to treat them with due respect, we bracket our own grievances and focus our attention on our beloved’s suffering rather than ourselves. By contrast, as much as the fanatic is concerned with the violation and misrecognition of their sacred values, they are concerned with the violation and misrecognition of themselves (Szanto 2022). The fanatical Muslim, for example, feels personally offended by cartoons of the prophet Mohammed, as does the fanatical US-American nationalist by the desecration of the US-American flag. Describing the fanatic as self-centered is not to say that they are concerned with *nothing but* themselves. It is to say that in caring about their values, they care about themselves as those who recognize and defend these values, and in caring about themselves, they care about the values they are committed to. As the example of “selfless” devotion shows, this is not the only possible way of caring about something. But it is not a rare condition either and it is certainly not restricted to fanaticism. To say that the fanatic’s fear is grounded in their double concern with their sacred value and their threatened self-respect is not an empirical claim about all people we call fanatics. Perhaps there are “selfless fanatics,” and perhaps the question of whether there are “selfless fanatics” is even one of the core controversies about fanaticism (Lerner 2019). Rather, it is to suggest a specific reading of fanaticism—a reading that, I suggest, helps us to better understand (and maybe even confront) the violent force of fanaticism but one that also has its limitations and, as such, needs to be complemented by other readings.

Third, the fact that the fanatic perceives harm, questioning, and dissent as attacks on both their values and themselves reveals that both their values and identity are fragile (Katsafanas 2019). The fanatic is vulnerable to what others think and do. By contrast, we can imagine other moral universalists who demand the universal recognition of their values, e.g., of human rights, and feel indignation in the face of human rights violations and yet feel so certain and secure in their commitment that even the most horrible human rights violations do not put into question their values and identity. The fanatic’s values and identity are fragile in that they are dependent on other people’s consent and acknowledgment. In itself, this is not necessarily a bad thing. If our best friend warns us that our new lover exploits rather than loves us, we might be better off listening to them even if this disturbs our sense of identity. Yet, while in the case of love we are dealing with highly particular values, the fanatic conceives of their values as universal. They not only require other people to acknowledge their values and corresponding way of life as one possible and legitimate one; they require all people to adopt the very same values and way of life and confront practical and theoretical dissent with violence. Only when one’s sense of fragility is answered with violence against the other who puts into question or violates one’s values and self-conception are we dealing with a form of fanaticism. It is the fanatic’s *flight from* fragility rather than their fragility itself that is problematic.

### Fraternity-Terror

In the previous subsection, I pointed out how, in the case of fanaticism (and other hostile antagonistic political movements), group antagonism is infused by the ingroup’s fear of outgroup dissent. I thereby presupposed that group antagonism is mainly a dyadic phenomenon. However, the reality is more complex; neither the ingroup nor the outgroup is homogenous—not even from the perspective of the fanatic themselves. First, if we conceive of fanaticism as a political strategy, it must be conceded that between ingroup and outgroup—“good” and “evil”—there is a “neutral” group that the fanatic seeks to mobilize in their struggle for what they take to be a sacred value (Olson 2007). Depending on how the fanatical group is defined, the group of potential recruits can be bigger or smaller. If it is exclusively defined in terms of shared values, anyone willing to commit to the values in question, and to live accordingly, is eligible for membership. But possible group membership can also be restricted by less fluid criteria such as religion, ethnicity, gender, or class. In any case, fanatical groups characteristically try to mobilize people eligible for group membership to actively join their struggle. *In this regard*, fanaticism is more appropriately described as a triadic rather than a dyadic structure. However, given that “neutrality” is an impossible stance with regard to sacred values (“you are either with us or against us”), “neutrality” appears as a form of evil. Being eligible to join the fanatical group becomes an obligation and the failure to do so becomes a form of complicity with evil (Berger 2018: 63). Therefore, fanaticism is neither a purely dyadic nor a purely triadic phenomenon, but rather characterized by a fluctuation between the two.

Second, there may arise a boundary within the fanatical group itself, demarcating a frontier between those who do and those who do not fully commit to and follow the fanatical group’s code of conduct. Questioning of, harm to, and dissent from the sacred values that define the fanatical group’s identity can be found not only outside but also within the fanatical group itself (Hoffer 2019: 152–154; Passmore 1990: 7). Group members can change their minds or for other reasons, such as akrasia or apathy, fail to live up to and defend their group’s shared values. Characteristically, this boundary that may arise within the ingroup is permeable. Although in some rare cases, the classification of group members may be clear and unalterable—the leader is good, the traitor is bad—the reason why the demarcation functions as a motivational incentive and as an instrument of terror is that, in principle, *anyone* can cross the boundary from the center to the margins of the group (although not necessarily the other way around). This makes the fanatic suspicious not only of their fellow fanatics but also, as I argue in the next section, of themselves. Even in groups that are primarily defined in terms of essentialized ascriptive qualities, it is not enough to simply *have* the qualities in question; they always come with prescriptive standards for how a member of the group *ought to* feel, think, behave, and act in order to remain a full member of the group. The claim that fanatical groups are characterized by a clear ingroup–outgroup demarcation accordingly needs to be revised: it is the *dialectic* of the clarity and (partial) permeability of borders that is characteristic of fanaticism.

The relationship with ingroup members who fail to live up to the fanatical group’s value commitments is characterized by hostility. In this regard, it resembles the fanatic’s relationship with members of the outgroup. However, it is a more ambiguous hostility than that towards the members of the outgroup because the other is still recognized as one of “us.” Again, this hostility can be explained by fear: a fear not of outgroup but ingroup dissent. From within, the fanatical group is constantly threatened by dissolution and collective akrasia. Here, I use the concept of collective akrasia in a loose sense to denote phenomena in which the group’s behavior diverges from its beliefs and commitments. While the threat of dissolution reflects the temporal character of human existence—people can *change* their value commitments and identity—the threat of collective akrasia reflects the basic structure of human will—our behavior and actions can diverge from what, according to our own practical reason, is the best thing to do.

To start with the first threat—namely that of dissolution—at first sight, ingroup doubt and dissent are threatening for the very same reasons as outgroup doubt and dissent. Any kind of doubt and dissent poses a threat to the fanatic’s sacred values, no matter whether it arises from without or within the fanatical group itself. On closer inspection, however, ingroup doubt and dissent may be even more threatening than the very same behavior attributed to the outgroup. This is the case because, first, although fanatics fear outgroup dissent, they also need dissenting others in order to uphold their sense of identity; in this regard, their ingroup’s identity is dialectically interwoven with that of the outgroup (Szanto 2022)—but not with that of dissenting members of the ingroup. Second, ingroup dissent directly puts into question the group’s identity and threatens the group with dissolution. Given that the group’s identity is defined in terms of the joint commitment to a shared sacred value, it is not simply a private matter when a group member fails to adhere to the value in question. It is also a social matter because it is not only a violation of a sacred value but also a violation of a joint commitment that as such directly puts into question the group’s identity and existence. More precisely, it first lowers the group’s self-esteem because it impairs the group’s capacity to realize its own values; second, it puts into question the group’s *descriptive* self-conception because it is no longer clear whether the group still *is* jointly committed to the sacred value in question; and third, it puts into question the group’s *evaluative* self-conception because the commitment to the value in question is a description under which the fanatical group values itself.

Again, the fanatic’s self-conception is characterized by fragility. The fanatical group is constantly threatened by dissolution. This time, the fragility is not answered with outgroup hostility but with constant suspicion with regard to one’s fellow fanatics (Hoffer 2019: 152–154) and “fraternity-terror” (Sartre 2004: 428–444)—i.e., the violent enforcement of group conformity that is a key feature of fanatical and extremist movements (Berger 2018: 63–64). Fraternity-terror is not a contingent feature of fanatical groups. Rather, it points back to the very construction of these groups themselves and in this sense is “ontological.” By becoming members of the fanatical group, fanatics jointly commit themselves to a sacred value. They grant the other the right to punish them if they fail to live up to their shared commitment. As Sartre (2004: 431) puts it, “[t]o swear is to say, as a common individual: you must kill me if I secede.”

Although the possibility of terror is grounded in the ontology of fanatical groups themselves, its actual implementation can vary depending on the circumstances. This leads me to the second threat mentioned above, namely that of collective akrasia. When the fear of the outgroup is sufficiently intense, it may not be necessary to appeal to fraternity-terror because the fear of the outgroup is enough to mobilize the fanatical group to extreme actions. However, when the fear of the outgroup decreases although the outgroup still constitutes an existential threat, fraternity-terror may help to maintain the necessary level of arousal to mobilize the fanatical group in defense of their common goal. “The regulatory third party reveals that the diminishing fear of danger is the real threat, and that it must be counteracted by increasing fear of destroying the group itself. The aim is the same: to protect the common interest. But, in the absence of any material pressure, the group *must produce itself as a pressure on its members*” (Sartre 2004: 430). This fear of the fanatical group’s collective akrasia is thus a “fear of the absence of the very fear that serves as the motor of … [the fanatical group’s] solidarity” (Toscano 2017: 303).

We are here pointed to a dialectic inherent to the fears of fanaticism. They are directed at threats arising from both within and without the fanatical group itself, and both kinds of threats are mutually dependent on each other. It is the outgroup that threatens the fanatic’s sacred values, but the size of the danger is inversely proportional to the fanatics’ fear of the outgroup. The more afraid the fanatics are of the threat imposed on them by the outgroup, the more willing they are to sacrifice themselves in combat. This, in turn, diminishes the danger imposed by the outgroup. The less afraid they are, the more dangerous becomes the unfelt threat imposed by the outgroup on account of it remaining unaddressed. It is the possibility of inner dissolution as well as the possibility of a discrepancy between felt threat and real danger (“real” from the perspective of the fanatical group, that is) that is reflected in the fanatical group’s self-reflexive anxiety—i.e., their fear that they themselves might fail to live up to their value commitments and, in doing so, fail to be or remain who they claim, desire, and value to be (Tietjen 2020).

### Self-Reflexive Anxiety

In the previous two subsections, I showed how fanaticism characteristically involves both fears of external and internal threats to the fanatic’s sacred values. Whereas “external” here meant threats arising from the outgroup, “internal” denoted threats arising from within the fanatical group itself. The latter phenomenon thus introduced some form of dialectic into the picture; the fanatical group is no longer seen as unambiguously good but rather becomes a battlefield on which evil needs to be fought in the form of the possible change and weakness of the will of one’s fellow fanatics that threaten the sacred value one is jointly committed to and, together with it, the fanatical group’s identity and very existence. However, this turn inwards needs to be taken one step further in order to understand the full dialectic of the fanatic’s fearfulness. While the threats I discussed in the previous section were “internal” in that they arose from within the fanatical *group*, they remained “external” in that they were attributed to *others* rather than to oneself. This points us to the third form of hostile antagonism involved in the phenomenon of fanaticism, namely self-hostility—i.e., a hostile antagonism inherent to the fanatic themselves.

The fanatic, as I have argued, feels their group to be constantly threatened by the possibility of both dissolution and collective akrasia. Others might deviate from the group’s shared values and in doing so threaten the values, the group’s identity, and its very existence. But it might also be oneself who deviates from the group’s shared values and, in doing so, threatens its values, identity, and existence. This presupposes that there is some form of ambiguity inherent to the fanatic themselves. Again, this might be an ambiguity between the fanatic’s past or present and their future self (i.e., a diachronic ambiguity): the fanatic is not sure whether their future self will stick to their present or past self’s value commitments. Or it might be an ambiguity inherent to their present self (i.e., a synchronic ambiguity): the fanatic feels that, although they are currently committed to the group’s sacred value, their commitment might be less than total. In any case, what the fanatic feels is a form of self-reflexive anxiety—i.e., they take an aversive stance towards who or what they as a person might be, might have been, or might become (on self-reflexive anxiety, see Tietjen 2020; on diachronic and synchronic ambiguity as characteristic features of fanaticism, see also Salmela and Capelos 2021). The fanatic’s self-reflexive anxiety reflects the fact that sacred values are not only endangered when *others* fail to recognize them but also when oneself fails to do so properly.

In this regard, the system of terror established by fanatical groups not only serves the purpose of protecting oneself and the group from *the other* but also of protecting the group and oneself from oneself *as the other*. “My ‘sworn faith’ is reflected to me *as a surety against my freedom*, through that of the third party: in fact it is this which gives him a real possibility of swearing, since it is because of it (and, of course, because of everyone else’s) that the possibility of relapsing into alterity no longer depends on him alone” (Sartre 2004: 423).

Ascribing self-reflexive anxiety to fanatics is not to make a claim about how all fanatics feel; it is not to point out a necessary feature of all fanatics. Rather, it is to make a claim about how fanaticism as a social phenomenon functions—namely, to silence dissent and ambiguity. With regard to individual fanatics—even those who seem supremely confident—this perspective calls us to look behind their seeming confidence and ask whether beneath it there might be a deep form of insecurity.

## Fragile Identities

In the previous section, I argued that the fears of fanaticism are dialectic in a twofold way. First, the threats they are directed at are simultaneously threats to the sacred value the fanatics are jointly committed to and to the fanatic group’s identity and existence itself. Second, the threats they are directed at arise from both within and without the fanatical group, from both within and without the fanatic themselves. I thereby discussed three forms of fear and anxiety connected to fanaticism: the fear of outgroup dissent, ingroup dissent, and inner ambiguity. In this section, I turn to a fourth form of fear or anxiety connected to fanaticism, namely the fanatic’s aversion to and flight from the existential condition of uncertainty itself. As I argue, at least in some cases, it is this anxious flight from uncertainty that grounds the fanatic’s fearfulness—i.e., their more specifically directed fears of outgroup dissent, ingroup dissent, and inner ambiguity.

Socio-psychological theories frequently cite uncertainty as a formation condition of fanaticism. In the first place, uncertainty describes the epistemic state of an agent who lacks full information about relevant issues. In order to become an antecedent of fanaticism, uncertainty needs to be *felt*. It is *affective* rather than epistemic uncertainty alone that is claimed to give rise to fanaticism. As a feeling, uncertainty ranges from diffuse to focused cases (Hogg 2007: 77). We can be uncertain about our future or about how to answer a specific question at a job interview; our uncertainty can reflect our personal situation (e.g., having lost our job) or the societal/global situation (e.g., uncertainty resulting from a global health crisis). Uncertainty is not always experienced as unpleasant. It can also be exhilarating and exciting, for example, when traveling to an unfamiliar country, taking up a new profession, or falling in love (Hogg 2007: 73). It is *aversive* uncertainty—i.e., uncertainty that is experienced as unpleasant and threatening—that may contribute to the emergence of fanaticism. While some of our uncertainties are about trivial matters, others concern matters of central import to our lives, such as our basic values and identity (Hogg 2007: 73), the world at large, or our place in the world (Proulx 2012: 55; van den Bos 2009: 198). It is these latter uncertainties that are potentially threatening and that, as such, may nourish fanaticism.

Uncertainty that is experienced as unpleasant and threatening motivates avoidance. There are two ways out of uncertainty. First, one can try to resolve or reduce uncertainty through epistemic enterprises such as deliberation or information gathering (Kurth 2018, p. 69; see also Nagel 2010 on epistemic anxiety). But this is a difficult enterprise, if not utterly unattainable given that aversive uncertainty can be understood not only as a reaction to a contingent lack of knowledge but also as a reaction to a more profound lack of knowledge characteristic of the human condition as such (Furtak 2019; Heidegger 1977; Lerner 2019; Tietjen 2021b). Alternatively—especially in situations that involve a sense of existential uncertainty—one can turn away from what one feels uncertain about and seek certainty in other domains of life—for example, in religion, the adoption of a middle-class lifestyle, or fanatical groups that provide one with a worldview and self-conception (allegedly) free of ambiguity and uncertainty. In this regard, fanaticism can be understood as an expression of the desperate need for sacred values (Hoffer, pp. 105–106). As uncertainty-identity theory claims, although “uncertainty about or reflecting on self can be resolved in many different ways, … group identification is one of the most potent and effective ways to do this” (Hogg 2007, p. 79). In a similar vein, the uncertainty management model argues that “under conditions of heightened personal uncertainty people’s cultures and the values they convey become especially important to them. Thus, they will value it particularly if persons or events adhere to the norms that are important in their culture or subculture, and they will hate it when people or events violate these norms” (van den Bos 2009: 203; see also McGregor and Marigold 2003).

What these theories claim is that in some cases, an aversion to uncertainty contributes to the emergence of fanaticism by motivating people to search for certainty in other, unrelated domains of their life. Moreover, they claim that group identification, and especially the identification with highly entitative groups, is particularly apt to provide the desperately sought-after sense of certainty or at least to *diminish* uncertainty. However, if my analysis of the fears of fanaticism is correct, then even the diminishment of uncertainty is illusory. Uncertainty continues to lurk behind the fanatic’s seemingly certain sense of identity; even worse, it is pushed to an even higher level, even if it is repressed and therefore not consciously experienced as such. Not only is the fanatic’s seemingly certain self-conception and worldview constantly haunted by uncertainty, but their illusion of certainty is also itself constantly under threat of being revealed for what it is, namely a flight from the existential condition of uncertainty itself.

The mechanism at stake here is similar to other psychic defense mechanisms such as *ressentiment* that help “individuals resist their insecurities and flaws without acknowledging or resolving them” (Salmela and Capelos 2021: 192). The repression of uncertainty, accordingly, creates a “fragmented” or “broken” self (Salmela and Capelos 2021: 194); insecurity and uncertainty are split off from the certain self that now knows what is right and wrong, good and bad. However, the mechanism of repression requires the constant investment of psychic energy in order to prevent the mechanism from breaking down. In this regard, the fanatic’s fragility, their fear of dissent and ambiguity, and their need for group confirmation can be interpreted as signs for their constant struggle to not relapse into their old, fragile sense of existence.

According to this picture, at least some fanatics experience uncertainty per se as threatening. Rather than answering uncertainty with epistemic behavior, they flee uncertainty and search for certainty in sacred values and group identity. Whereas sacred values provide them with certainty about the world, group identity solidifies their sense of self. This construct, however, itself is fragile. This explains why violence against people holding alternative worldviews or living different lives, for the fanatic, fulfills both an expressive and a performative function. It *expresses* the fanatic’s value convictions on the level of intentional content and attitude and, at the same time, it *performatively brings about* the very same attitude.

## Outlook

The description of the fears of fanaticism as a flight from the existential condition of uncertainty itself suggests that everything depends on how we cope with (existential) uncertainty: whether we experience it as unpleasant or exhilarating, whether we flee from it or embrace it. Psychological theories tell us that aversion to uncertainty can take two different forms depending on what kind of concern it is based on (Hogg 2007: 77–78). People whose primary concern is epistemic—they want to be *right*—are primarily aversive to invalidity; in them, uncertainty motivates epistemic behavior. By contrast, people who are primarily aversive to uncertainty itself—they want to be *certain*—are primarily concerned with resolving uncertainty quickly and/or permanently—even at the expense of truth and knowledge gain (Kruglanski 2004; Kruglanski and Webster 1996).

This might conciliate us and give us hope, if not with regard to *others*—those we call “fanatics”—then at least with regard to ourselves—philosophers and other researchers who, in their scientific and philosophical explorations, believe themselves to be committed to truth rather than certainty. Nietzsche (1999, 2000), however, warns us that this *certainty*—how else should we call it?—Might itself be illusory at best and fanatical at worst because what if our obsession with truth is itself a result of the very same psychological mechanism as the fanatic’s commitment to *their* sacred value? These considerations point us back to the very beginning of this article and remind us that it is not possible to simply bracket the socio-political structures in which our discourse and theorizing about fanaticism are embedded. The concept of fanaticism is highly pejorative and almost exclusively applied to others rather than to oneself. In this regard, the discourse on fanaticism is itself characterized by a strong form of othering that draws a clear line between those who are fanatics (“them”) and those who are not (“us”) and claims to have a clear knowledge about how to draw the boundary between these two groups that is backed up by scientific knowledge. A critical philosophical theory of fanaticism needs to take seriously the danger of fanatical intolerance and violence but also the violent (e.g., unjustifiably exclusionary) potential of our discourse on fanaticism. While not denying their difference, it must take seriously the dialectic of fanatical and anti-fanatical violence. Applied to the subject matter of this article, this means that an effective theory of fanaticism needs to complement the analysis of the uncertainties, fears, and anxieties that drive fanatics with an analysis of the fearful attitudes toward fanaticism that potentially underlie and ground our critical stance toward fanaticism.

For the question of how to avoid fanaticism this implies that, although important, replacing the search for certitude with a search for truth will be unlikely to solve the problem because both endeavors rely on the same presupposition, namely an aversive stance toward uncertainty itself. Especially when it comes to existential uncertainty, it might be more promising to counterbalance the (apt, yet incomplete) socio-politically powerful depiction of uncertainty as a threat with depictions that foreground the liberatory potential of uncertainty and ambiguity. Depictions that satisfy these needs, in turn, will more likely fall into the domain of the arts rather than the sciences. In this regard, one of the unique credentials of philosophy in thematizing and confronting fanaticism is that it can be practiced as both a form of science *and* a form of art.
